# Alpha-Band Lateralization and Microsaccades Elicited by Exogenous Cues Do Not Track Attentional Orienting

**DOI:** 10.1523/ENEURO.0076-23.2023

**Published:** 2024-02-06

**Authors:** Elio Balestrieri, René Michel, Niko A. Busch

**Affiliations:** ^1^Institute for Biomagnetism and Biosignal Analysis, University of Münster, Münster 48149, Germany; ^2^Otto-Creutzfeldt-Center for Cognitive and Behavioral Neuroscience, University of Münster, Münster 48149, Germany; ^3^Institute of Psychology, University of Münster, Münster 48149, Germany

**Keywords:** alpha oscillations, exogenous attention, inhibition of return, microsaccades

## Abstract

We explore the world by constantly shifting our focus of attention toward salient stimuli and then disengaging from them in search of new ones. The alpha rhythm (8–13 Hz) has been suggested as a pivotal neural substrate of these attentional shifts, due to its local synchronization and desynchronization that suppress irrelevant cortical areas and facilitate relevant areas, a phenomenon called alpha lateralization. Whether alpha lateralization tracks the focus of attention from orienting toward a salient stimulus to disengaging from it is still an open question. We addressed it by leveraging the phenomenon of inhibition of return (IOR), consisting of an initial facilitation in response times (RTs) for stimuli appearing at an exogenously cued location, followed by a suppression of that location. Our behavioral data from human participants showed a typical IOR effect with both early facilitation and subsequent inhibition. In contrast, alpha lateralized in the cued direction after the behavioral facilitation effect and never re-lateralized compatibly with the behavioral inhibition. Furthermore, we analyzed the interaction between alpha lateralization and microsaccades: while alpha was lateralized toward the cued location, microsaccades were mostly oriented away from it. Crucially, the two phenomena showed a significant positive correlation. These results indicate that alpha lateralization reflects primarily the processing of salient stimuli, challenging the view that alpha lateralization is directly involved in exogenous attentional orienting per se. We discuss the relevance of the present findings for an oculomotor account of alpha lateralization as a modulator of cortical excitability in preparation of a saccade.

## Significance Statement

Our exploration of the surrounding visual environment relies on constant attentional shifts toward different spatial locations, tightly coupled to moment-to-moment changes in electrical brain activity. One of the most prominent signatures of this activity, the alpha oscillations, has long been considered a central substrate of these attentional shifts. Here we show that alpha oscillations, albeit being correlated to the direction of attention toward a salient spatial position, do not track the disengagement from this position. Moreover, we found that alpha oscillations are tightly related to miniature eye movements. Altogether, these findings challenge the notion that alpha oscillations are a fundamental substrate of attentional shifts and suggest that alpha oscillations subserve a preparatory function for eye movements.

## Introduction

The most salient neuronal signal in the human electroencephalogram is the alpha rhythm—an oscillatory pattern over the occipital cortex with a characteristic frequency of 10 Hz. The topographical distribution of alpha oscillations is correlated with spatial attention, such that alpha power is reduced in the hemisphere contralateral to the attended location relative to the ipsilateral hemisphere. This lateralization is usually induced with cues indicating a task-relevant location and has been observed in a variety of paradigms including endogenous cueing (Worden et al., 2000; Busch and VanRullen, 2010; Harris et al., 2018), exogenous cueing (Feng et al., 2017; Keefe and Störmer, 2021), visual working memory (VWM; Sauseng et al., 2009; Boettcher et al., 2021), and visual search (Bacigalupo and Luck, 2019). Collectively, these findings demonstrate that the direction and timing of alpha lateralization coincides with the selection of task-relevant spatial locations. According to a long-standing interpretation, cue-induced alpha lateralization reflects the active regulation of cortical inhibition, whereby processing in task-relevant areas (e.g., the occipital cortex contralateral to visual stimulation) is protected from distraction by suppressing task-irrelevant areas (Jensen and Mazaheri, 2010; Foxe and Snyder, 2011).

However, a number of studies have found evidence conflicting with a direct causal involvement of alpha lateralization in cue-related suppression: while alpha lateralization follows the task-relevant location, it is often not affected by the location of irrelevant or distracting stimuli (Noonan et al., 2016; Schroeder et al., 2018; Foster and Awh, 2019; van Moorselaar and Slagter, 2019; Pietrelli et al., 2022). Moreover, Antonov et al., (2020) compared the dynamics of alpha lateralization to that of steady-state visual evoked potentials (SSVEPs), an established neuronal marker of stimulus processing in the early visual cortex. As expected, SSVEPs were amplified at attended locations, but alpha lateralization toward those locations lagged behind the SSVEP effect. Moreover, while alpha power increased in the hemisphere representing the unattended location, this was not associated with SSVEP suppression (Antonov et al., 2020; Gundlach et al., 2020). Collectively, these findings suggest that alpha lateralization might be a secondary consequence of cueing and spatial selection. Likewise, spatial cueing is often associated with microsaccades—miniature eye movements that occur even when subjects are instructed to keep fixating. Critically, the occurrence and direction of microsaccades is influenced by endogenous (Engbert and Kliegl, 2003) and exogenous cues (Galfano et al., 2004; Rolfs et al., 2004; Lv et al., 2022). Interestingly, recent findings show a strong interdependence between local changes in alpha power and microsaccades (Liu et al., 2022a) or eye movements in general (Popov et al., 2021). As a consequence, a characterization of alpha lateralization in response to a spatial cue has to take into account also the interplay between microsaccades and alpha lateralization.

In the present study, we tested the hypothesis that alpha lateralization is a consequence of cue processing that is not directly involved in attentional orienting. Most previous studies have been designed to induce attentional orienting at the cued location, making it impossible to distinguish cue processing and orienting. Here, we leveraged the inhibition of return (IOR) phenomenon, where attention is oriented away from an exogenously cued location (Posner et al., 1985; Klein, 2000). In a typical Posner task, in which an exogenous, uninformative cue is followed by a target after a variable delay, reaction times show a robust two-stage pattern: short-lived facilitation at short delays with faster responses to targets at the cued position followed by long-lasting inhibition at delays longer than ∼220 ms with faster responses at the uncued position (Samuel and Kat, 2003). This pattern is believed to reflect the time course of attentional deployment, with an initial orientation toward the cued position, and subsequent disengagement and re-orientation toward the uncued position (Klein, 2000; Klein and Hilchey, 2011). Accordingly, if alpha lateralization reflects spatial orienting, the time course and direction of alpha lateralization should mirror the behavioral effect of IOR, by showing an initial facilitation for the cued location (increase in alpha power ipsilaterally, decrease contralaterally), followed by a pattern reversal corresponding to the inhibition of the cued position. Alternatively, if alpha lateralization were only a secondary consequence of attentional orienting associated with cue processing, the direction of lateralization should only reflect the cued location. Moreover, given that the typical exogenous Posner cueing task is accompanied by microsaccades away from cued position at approximately the onset of the IOR (Galfano et al., 2004, Rolfs et al., 2004, Lv et al., 2022), we also aimed to characterize the interaction between cue-induced shifts in alpha lateralization and microsaccades.

## Materials and Methods

### Pre-registration and participants

The present study was conducted and analyzed as described in the pre-registration (aspredicted.org), unless stated otherwise. We had planned to collect data from 20 participants after exclusion based on the following: (1) poor behavioral performance (false alarm rate, FAR > 0.20), (2) incomplete sessions, and (3) poor EEG data quality (*n* trials rejected for artifact, >15%). We recorded data from 23 participants. Data from three participants were excluded due to high FAR and data from one participant were discarded due to poor eye-tracking data quality, a criterion that had not been pre-registered. The final sample was composed of 19 participants (*F*, 15; *M*, 4; mean age, 22.5 years; age range, 19–28). All participants gave written consent for their participation, reported normal or corrected to normal vision, and were compensated either with money (8 EUR/h) or with course credits. The study was approved by the ethics committee of the University of Münster.

### Apparatus

Recordings took place in a dimly lit, sound-proof cabin. Participants placed their heads on a chin-rest and could adjust the height of the table to be seated comfortably. Stimuli were generated using MATLAB 2019a (mathworks.com) and Psychtoolbox (Brainard, 1997; Kleiner et al., 2007). The experiment was controlled via a computer running Xubuntu 16.04, equipped with an Intel Core i5-3330 CPU, a 2 GB Nvidia GeForce GTX 760 GPU, and 8 GB RAM. The experiment was displayed on a 24″ Viewpixx/EEG LCD monitor with 120 Hz refresh rate, 1 ms pixel response time (RT), 95% luminance uniformity, and 1,920 × 1,080 pixels resolution (vpixx.com). Distance between participant eyes and the monitor was ∼86 cm. Eye movements were monitored using a desktop-mounted EyeLink 1000+ infrared-based eye-tracking system (sr–research.com) set to 500 Hz sampling rate (binocular). EEG was recorded with a Biosemi Active Two EEG system with 65 Ag/AgCl electrodes (biosemi.nl), set to 1,024 Hz sampling rate. Sixty-four electrodes were arranged in a custom-made montage with equidistant placement (“Easycap M34”; easycap.de), which extended to more inferior areas over the occipital lobe than the conventional 10–20 system (Oostenveld and Praamstra, 2001). An additional external electrode was placed below the left eye.

### Stimuli and experimental procedure

**Figure 1.**
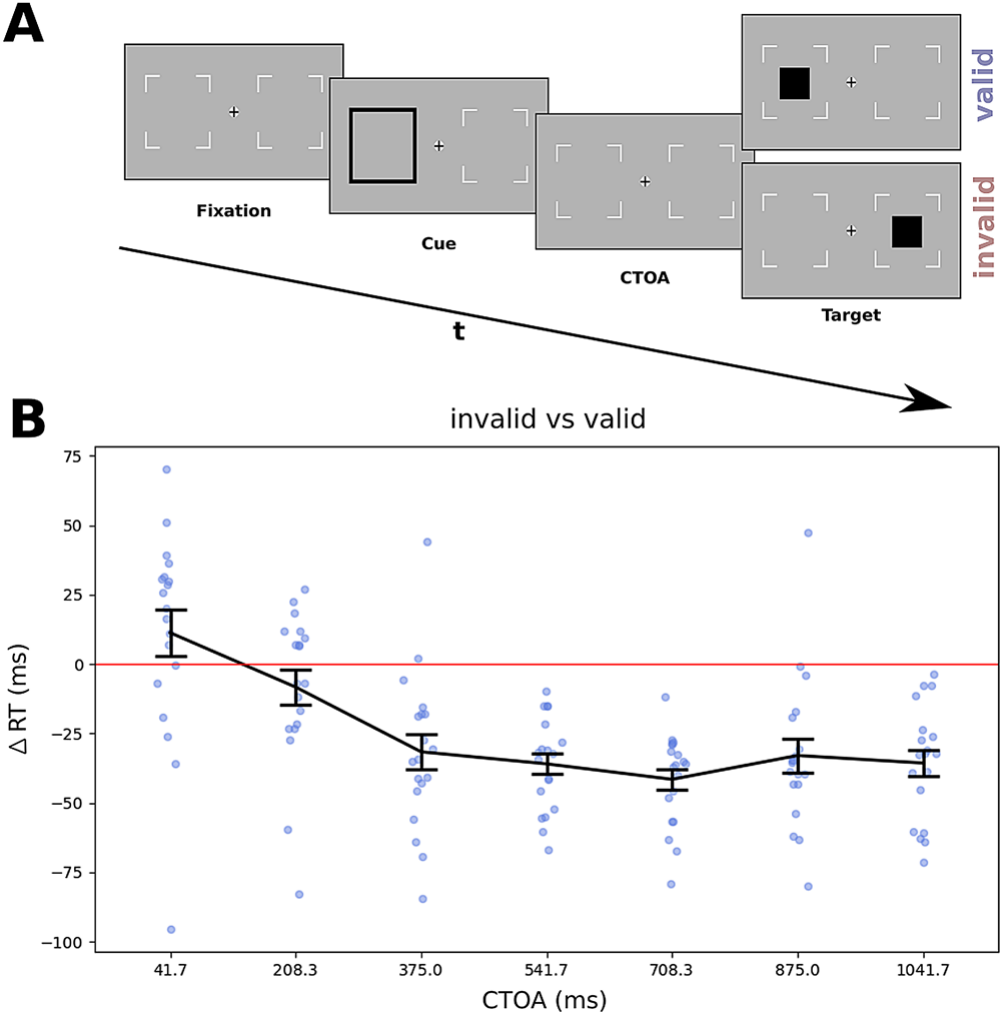
***A***, Illustration of stimuli and procedure. Every trial started with a fixation cross-flanked by two placeholders. After a jittered time interval, an exogenous cue (the black contour) flashed for 100 ms at one of the two positions. After a variable CTOA, a target (a black square) appeared for 33 ms either at the cued (valid) or uncued (invalid) position, and participants had to detect it by a speeded button press on a gamepad. ***B***, RT difference between invalid and valid cues as a function of CTOA; positive values indicate facilitation, and negative values indicate inhibition of the cued position. Error bars represent SEM.

For an overview of the stimulus arrangement, see Figure 1*A*. All stimuli were presented on a medium gray background (52.2 cd/m^2^). Two placeholders indicating target locations were positioned at 8 dva to the left and right of the central fixation marker (diameter = 1 dva; black and white; 0.2 cd/m^2^ and 102.3 cd/m^2^; Thaler et al., 2013). The cue was an amplification of one of the two target location placeholders, that is, thicker and darker (16.6 cd/m^2^; line width = 0.08°). The target was a dark square (diameter = 0.1°; 23.7 cd/m^2^) centered in one of the two target locations. After the target, only the placeholders and the fixation cross were presented until the response. A small colored rectangle (side = 0.2 dva) presented in the center of the screen served as feedback at the end of a trial (yellow for correct, blue for incorrect responses).

The study comprised a single recording session of ∼1.5 h duration to collect a total of 980 trials. Prior to the experiment, 10 to 30 practice trials were presented. Each session was divided into seven blocks of 140 trials, separated by breaks (self-paced, but at least 30 s), with each block containing a small break (self-paced, but at least 10 s) after 70 trials. The time course of IOR was sampled through seven cue–target onset asynchronies (CTOAs) ranging from 42 to 1,050 ms in steps of 168 ms with 28 trials per CTOA and validity condition (784 trials in total). Additionally, 196 catch trials (20%) without a target (from hereafter “no target” condition) were presented, equally distributed across blocks. Cue position, target position, and CTOAs were counterbalanced within each block.

The trial sequence is illustrated in Figure 1*A*. Each trial started with a fixation cross and two placeholders for 800–1,200 ms (randomized across trials), followed by a noninformative visual cue (cue validity, 50%) flashed for 100 ms. Except for no-target trials, the target was presented after a variable CTOA for 33 ms either at the cued location (valid) or uncued location (invalid). Both placeholders and the fixation marker remained on-screen until the participant's response. Participants were asked to press a button as fast and accurately as possible within 2 s whenever they detect a target regardless of its location. After response, participants received feedback indicating whether their response was correct. Subsequently, a blank screen was presented as intertrial interval for a random duration from 1,000 to 1,500 ms.

Correct fixation was monitored online, and trials in which the gaze drifted away from the central fixation marker for >2 dva were interrupted and repeated at the end of the block. The same procedure was applied in trials containing button presses before the target appeared on screen. A second fixation assessment was performed offline, leading to the exclusion of an average of 0.2% (SD = 0.3) of trials in which the online fixation control was inaccurate.

### Behavioral analysis

Data preprocessing was implemented in R 4.1.3. For each participant, RTs below 200 ms and above 4 SD were excluded from further analyses. RTs were *z*-scored based on the mean and standard deviation across all trials, and the time course of IOR was computed by averaging RTs separately for every CTOA and validity condition. Statistical analyses were implemented in Python 3.8 (python.org) and Pingouin (pingouin-stats.org), using a repeated-measures ANOVA with the within-factors validity (valid, invalid) and CTOA.

Plots representing the evolution of RTs across CTOAs show the difference between invalid and valid trials, so that positive values indicate facilitation (shorter RTs) for the cued position and negative values indicate inhibition (longer RTs).

### EEG preprocessing

EEG preprocessing was performed with EEGLAB (Delorme and Makeig, 2004) and custom scripts running on MATLAB 2021a (mathworks.com). Data were first downsampled to 256 Hz and re-referenced to common average. The continuous data were then high-pass filtered at 0.8 Hz and low-pass filtered at 40 Hz and then epoched from −1,000 to 2,000 ms time-locked to cue onset. A first set of trials were rejected based on extreme amplitudes (±500 µV) or on joint probability (function *pop_jointprob* in EEGLAB, local threshold 9, global threshold 5). ICA was then performed, and the IClabel algorithm (Pion-Tonachini et al., 2019) was used to classify components in the categories “Brain,” “Muscle,” “Eye,” “Heart,” “Line Noise,” “Channel Noise,” and “Other” based on their spatial topography. Components belonging to nonbrain sources with a probability above 0.5, as well as components labeled as “Brain” with a probability below 0.1, were excluded. After the artifactual components had been removed, noisy channels (whose SD > 3.5) were spherically interpolated. Due to good data quality after preprocessing, only one channel in one subject needed interpolation. Finally, remaining trials including amplitudes larger than ±150 µV were rejected (on average 2.6% of trials per subject; SD = 3.4).

### EEG data analysis

**Figure 2.**
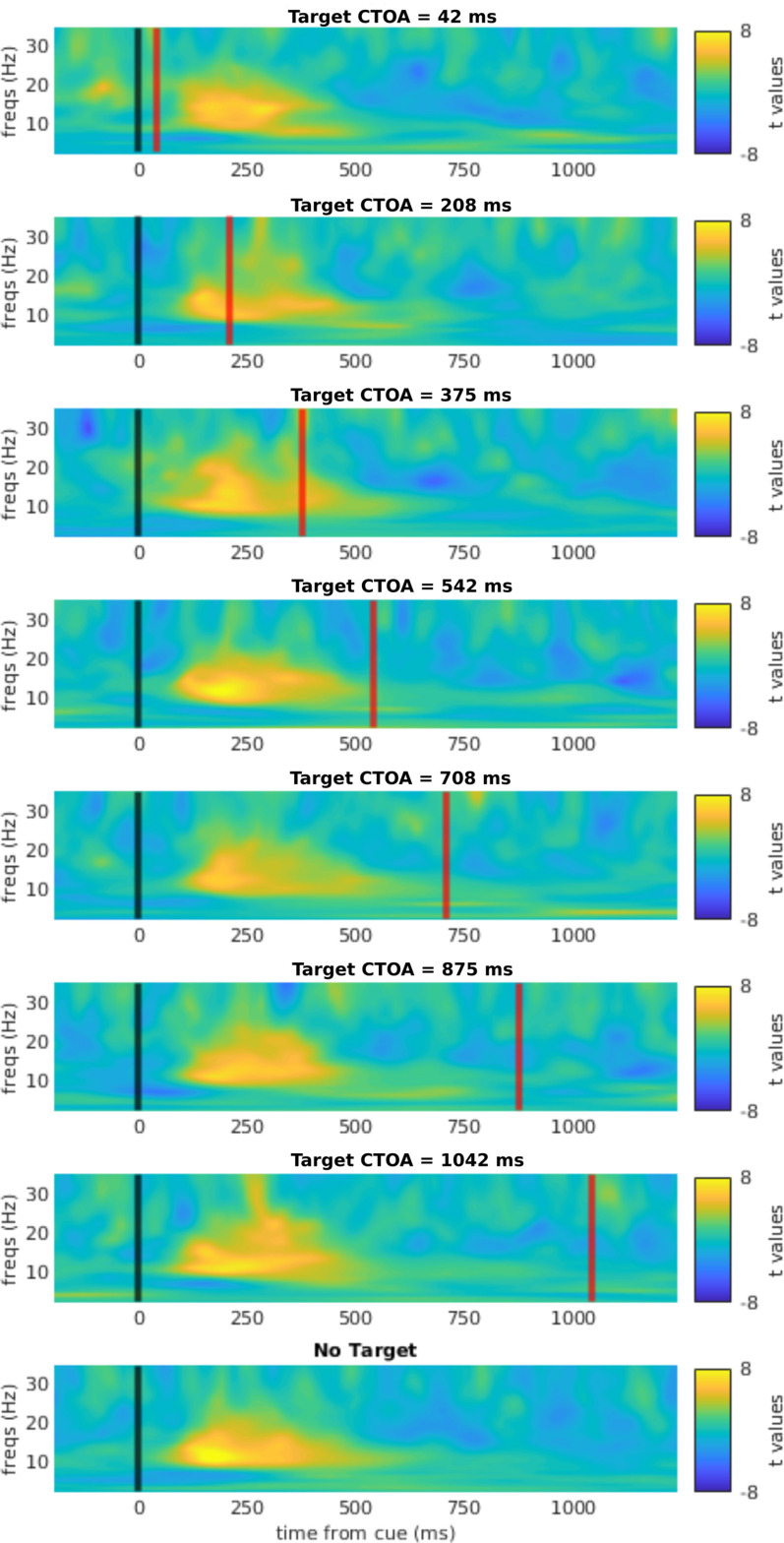
Time–frequency representation of lateralized power in response to the exogenous cue, collapsed across validity conditions. Note that the averaged ERP had been subtracted from the data before time frequency analysis, thereby removing the signals evoked by cue and target onsets. In all subplots, the black line at 0 indicates cue onset, whereas the red line indicates the target onset (except for no-target trials).

The analysis aimed to investigate the dynamics of exogenous orienting and re-orienting indicated by lateralized alpha oscillations. Given that both cues and targets were themselves lateralized stimuli, and given that stimulus-evoked potentials comprise substantial power at alpha frequencies (Schürmann and Başar, 2001), it was necessary to remove these stimulus-evoked responses from the signal. To this end, the average across trials (i.e., the evoked potential) was subtracted from each single trial prior to the time–frequency analysis, separately for each cue position × target position × CTOA condition. Thereby, this analysis ensures that the remaining alpha lateralization is not confounded by the cue-evoked or target-evoked sensory response (Klimesch et al., 1998). As a result, no alpha-band lateralization is visible following target onset in Figure 2, underlying the success of this procedure.

The time frequency analysis was performed using a wavelet transform with 34 wavelets over a frequency range between 2 and 35 Hz, full-width at half-maximum (FWHM) = 2/*f* (Cohen, 2019).

Lateralized power was computed as follows:

$$\mathrm{Index}=\frac{\mathrm{Powe}r_{\mathrm{left}}-\mathrm{Powe}r_{\mathrm{right}}}{\mathrm{Powe}r_{\mathrm{left}}+\mathrm{Powe}r_{\mathrm{right}}},$$

where Power is the spectral power (averaged across trials) for each frequency, in the left or right ROI. The ROIs for computing lateralization were two homologous subsets of five left and right occipital channels already shown to maximize alpha lateralization in a previous study (Balestrieri and Busch, 2022). Cue-induced lateralization was then computed as the difference in lateralization index between left and right cued trials. Thus, conventional cue-induced alpha lateralization would take positive values, indicating stronger power ipsilateral versus contralateral to the cue.

The bulk of the EEG analysis focused on the no-target condition, for which the entire post-cue interval was guaranteed to be free of any target-evoked signals. Alpha-band lateralization was further quantified by averaging across frequencies from 8 to 13 Hz. Cue-evoked lateralization was tested for significance with *t* tests against 0, under the null hypothesis of lack of any lateralization. We corrected for multiple comparisons through cluster permutation tests (see Cluster permutation tests).

### Microsaccades

Analyses of microsaccades had not been planned a priori and were hence not included in the original pre-registration. As for alpha lateralization, only no-target trials were analyzed to avoid target-related confounds. The eye-tracking data in X–Y screen coordinates from both eyes were averaged and microsaccades were detected using the function *pop_detecteyemovements* from the EYE-EEG toolbox (Dimigen et al., 2011) using the microsaccade detection algorithm by Engbert and Mergenthaler (2006). Lambda was set to 5, and the minimal microsaccade duration was set to three samples (11 ms). Smoothing of raw data was implemented by taking the moving average of velocities over five data sample, as in Engbert and Kliegl (2003). Furthermore, all eye movements exceeding 1 dva were excluded. Microsaccades in the direction of the cued location were labeled as “toward” and microsaccades in the opposite direction were labeled as “away.” Microsaccadic rate was computed with a linear moving average of 200 ms. As a final step, we computed a metric of microsaccadic rate bias (from hereafter “MS-bias”), as the difference between the rate of microsaccades toward and away from the cue.

### Mutual relationship between alpha lateralization, RTs, and MS-bias

We reasoned that if there was any connection between alpha lateralization, RTs, and MS-bias, they should covary across time. Hence we computed time point-by-time point Pearson’s correlations between these variables based on the no-target condition. Alpha lateralization and MS-bias were only quantified for no-target trials. Furthermore, alpha lateralization and MS-bias were subsampled to adjust for the sparse sampling of RTs (i.e., with just seven CTOAs) as follows.

Alpha lateralization was averaged across 100 ms long time windows, each starting 80 ms after each corresponding CTOA. We reasoned that target processing would be most affected by the magnitude of alpha lateralization at the approximate latency of target-processing in early visual cortex (Iemi et al., 2019), as opposed to target appearance on the screen. MS-bias was averaged across 100 ms long time windows immediately preceding each corresponding CTOA. Here, we reasoned that target processing would be most affected by the presence of microsaccades (and their direction) immediately before target onset. Pairwise correlations between alpha lateralization, MS-bias, and RTs were computed for all latency pairs to analyze for each signal at a given CTOA how and when this signal was associated with any of the other signals (e.g., whether alpha lateralization at the earliest CTOS was associated with RTs at later CTOAs). We also computed a sample-by-sample correlation between alpha lateralization and MS-bias using the densely sampled data, before binning it in the seven CTOAs. In all the aforementioned analyses, results were corrected for multiple comparisons via cluster permutation.

### Cluster permutation tests

Cluster permutation tests were used to correct for multiple comparisons. For the tests involving correlations between different time series, we used the procedure detailed by Kluger et al. (2021). The procedure was analogous for (1) correlations between RTs and MS-bias rate, (2) RTs and alpha lateralization, and (3) alpha lateralization and MS-bias. For each time point, we correlated the vector of intersubject values belonging to the first time series (e.g., alpha lateralization) with the paired vector of the other time series (e.g., MS-bias). This yielded a set of Pearson’s *r* and corresponding *p* values. In order to correct for multiple comparisons, we applied a cluster permutation approach as follows. We first detected the significant clusters in our data and obtained the cluster masses by summing together the *r* values of the contiguous significant points in time (*p* < 0.05). Then we reiteratively shuffled (*N* = 1,000) the participant order in both the time series under test. For each iteration, we stored the absolute highest cluster mass value, yielding a surrogate distribution of cluster masses. We rejected H0 for each cluster mass in the real data that was below the 2.5th or exceeded the 97.5th percentile (*α* = 0.05; two-tailed) of the surrogate distribution of cluster masses.

For the test against 0 used for assessing lateralization, or MS-bias, we also implemented cluster permutation tests ad hoc. *T* tests against 0 were performed for the whole time course. We defined clusters by selecting those *t* values contiguous in time and exceeding the critical threshold (*α* = 0.05) on both tails. From each cluster, we obtained a cluster statistic by summing together all the *t* values belonging to the cluster, that is, characterized by contiguity in space (electrodes) and time. To correct for multiple comparisons, for 1,000 repetitions, we multiplied a random participant’s subset by −1 and repeated the aforementioned steps in order to obtain a distribution of cluster statistics under the null hypothesis. We rejected the null hypothesis if a cluster statistic was below the 2.5th or above the 97.5th percentiles of the permutations.

### Bayesian analysis

We employed a Bayesian analysis to substantiate conclusions about the presence or absence of statistical effects. Bayesian statistics allows in fact to interpret the amount of evidence both in favor and against the null hypothesis rejection (Kass and Raftery, 1995). In this context, we opted to use the Bayes factor $BF_{10}$, defined as the predictive updating factor which measures the change in relative beliefs about hypothesis H1 relative to the null H0 given the data (Kelter, 2020). In particular, we aimed at gathering the amount of evidence in favor or against the null hypothesis for two sets of analyses: (i) the *t* tests against 0 showing directionality of effects in RTs, alpha lateralization, and MS-bias and (ii) the paired correlations between RTs and alpha lateralization, RTs and MS-bias, and alpha lateralization and MS-bias. We performed each *t* test and correlation for each one of the CTOAs probed, as detailed in the previous paragraphs. $BF_{10}$ was interpreted according the typical classification scheme (Kelter, 2020) that quantifies the amount of evidence on a log scale: in a nutshell, a $BF_{10}$ above 3 indicates moderate evidence for H1, whereas a $BF_{10}$ below 0.33 indicates moderate evidence for H0. $BF_{10}$ was computed via the bayesFactor package for MATLAB (Krekelberg, 2023).

### Single-trial alpha lateralization analysis

In order to gauge whether measures are associated with within-participant variations, for every participant, CTOA condition, and trial, we performed a time point-by-time point regression between alpha lateralization and RTs. And since the single-trial EEG activity is inherently noisy, we expanded our analyses based on ROIs with a more advanced method, namely, the effect-matched spatial filter, or EMS (Schurger et al., 2013), to determine a precise time course of cue-induced alpha lateralization at the single-trial level.

As a first step, for each participant, we computed a spatial filter based on the topographical distribution of alpha power (8–13 Hz) in the difference between left and right cue. This was performed in the condition without targets, for the time window (≈200–400 ms post-cue) where the alpha lateralization was the strongest. Figure 6*A* (top left inset) shows the average topography across all participants.

Secondly, always at the single-trial level, we computed a dot product between the alpha power across all the channels and the spatial filter for each time point, from the pre-cue (−200 ms) up until 1,500 ms after the cue. This was done for all the conditions with targets, for each CTOA. Importantly the sign of the template was adjusted according to the cued side, making the time series sign easily interpretable: positive for lateralization toward the cue and negative instead for lateralization in the opposite direction.

Thirdly, separately for valid (cue and target in the same position) and invalid (cue and target in opposite positions), we performed a time point-by-time point linear regression between alpha lateralization and *z*-scored RTs, separately for each CTOA condition. The RTs were *z*-scored across all experimental conditions (CTOAs) in order to preserve the intercondition variability intrinsic to the IOR. Concurrently, the regression model included an intercept, to account for differences across conditions independent from alpha lateralization.

As a last step, in each CTOA condition, we grouped the regression beta scores obtained in each time point for the single participants separately according to the cue–target validity, which we then tested against 0 with a cluster permutation test. Based on the alleged inhibitory effect of alpha oscillations (Jensen and Mazaheri, 2010; Foxe and Snyder, 2011), it is possible to hypothesize that, depending on cue validity, the pattern of lateralization with respect to the cue would show different correlations with RTs, graphically illustrated in Figure 6*B*. In the valid condition, a pattern of lateralization oriented toward the cue would predict faster RTs, whereas a pattern of lateralization away from the cue would predict slower RTs, leading to a negative correlation. On the contrary, in the invalid condition, a pattern of lateralization away from the cue would lead to fast RTs, whereas a pattern of lateralization toward the cue would be associated to slow RTs and, altogether, to a positive correlation.

## Results

### Behavior

To confirm the existence of an IOR effect, we subjected the RTs to a repeated-measures ANOVA with the within-factors validity (valid, invalid) and CTOA. The RT difference between invalid and valid conditions as a function of CTOA is shown in Figure 1*B*.

The pattern shows the typical initial facilitation and subsequent inhibition at the cued location and was confirmed statistically by the ANOVA, which showed on top of the main effect of validity (*F*_(1,18) _= 36.103; *p* < 0.001; *η*^2 ^= 0.667) and of CTOA (*F*_(6,108) _= 26.634; *p* < 0.001; *η*^2 ^= 0.597) the expected strong validity × CTOA interaction effect (*F*_(6,108) _= 17.431; *p* < 0.001; *η*^2 ^= 0.492). For each CTOA, we tested the difference between invalid and valid conditions via one-tailed paired samples *t* tests. The choice on one-tailed tests was justified by the specific directionality expected: shorter RTs for the validly cued position at the first CTOA and longer RTs for all the others. The first CTOA showed a trend toward significance (*t*_(18) _= 1.709; *p*_(uncorrected) _= 0.052), whereas all the other CTOAs after 225 ms (i.e., the typically observed onset of IOR) showed a strong advantage for the uncued position (all *t* < −5.729; all *p* < 0.001; Bonferroni’s corrected). This pattern of results shows that we successfully elicited IOR in our participants.

### Spectral and temporal pattern of cue-induced lateralization

We characterized the temporal and spectral profile of lateralized responses to the spatial cues, as presented in Figure 2. For each CTOA and for the no-target trials, we computed the lateralization index for the whole time window of possible target appearance (up to 1,050 ms post cue). In all conditions, cue-induced lateralization “toward” the cued location ramped up at ∼100 ms after cue onset and leveled off at ∼600 ms. This response was most prominent in the alpha band, but extended to higher frequencies in the beta band. This pattern was observed consistently for all CTOAs and both validity conditions, confirming that our exogenous cueing paradigm successfully elicited a relative increase in power at ipsilateral channels relative to the cue. However, we did not observe any re-lateralization “away” from the cue at longer latencies that would mirror the behavioral IOR effect at longer CTOAs. This mismatch between patterns of behavioral data and lateralized power was investigated more systematically in the subsequent analyses.

### Time course of alpha lateralization and its relation to RTs

**Figure 3.**
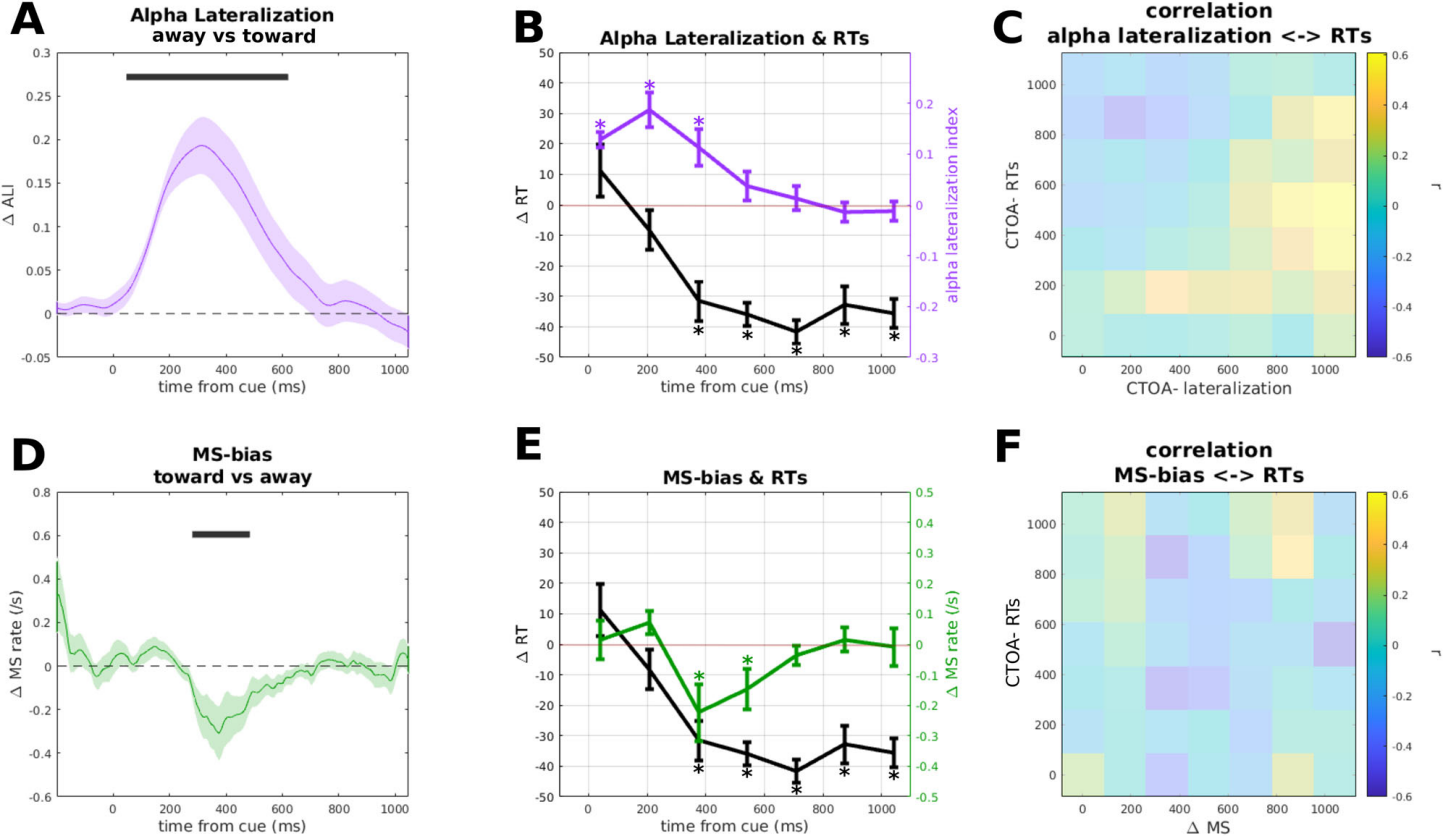
***A***, Lateralization in the alpha band (8–13 Hz). Positive sign indicates increase in alpha ipsilaterally and decrease contralaterally. The dark gray bar indicates the significant cluster tested against 0. Shaded area represents SEM. ***B***, Comparison between RTs and alpha lateralization (downsampled to match the RT time course; see Materials and Methods). The different color-coded *y*-axes are set to different scales to allow a direct comparison between alpha lateralization and RTs. Error bars represent SEM. Asterisks indicate significant *t* tests against 0. ***C***, Intersubjects correlation matrix between alpha lateralization and RTs. The transparency masking indicates that no test survived correction for multiple comparisons. ***D***, MS-bias, toward (positive) and away (negative) from the cued location. The dark gray bar indicates the significant cluster tested against 0. Shaded area represents SEM. ***E***, Comparison between RTs and MS-bias (downsampled to match the RT time course; see Materials and Methods). The different color-coded *y*-axes are set to different scales to allow a direct comparison between MS-bias and RTs. Error bars represent SEM. Asterisks indicate significant *t* tests against 0. ***F***, Intersubjects correlation matrix between MS-bias difference and RTs. The transparency masking indicates that no test survived correction for multiple comparisons.

Given that our a priori hypothesis targeted the alpha band, which was also the band with the strongest lateralized effect in the current analysis, we focused on the power averaged in the 8–13 Hz range and restricted the analysis to no-target trials, which were unconfounded by any target-evoked signals. The resulting time course showed pronounced and sustained alpha lateralization toward the cue position (i.e., increase ipsilateral and decrease contralateral to cue) starting at cue onset, peaking at ∼250 ms and fading away after 650 ms (cluster *p* < 0.001; Fig. 3*A*). Importantly, lateralization did not invert *away* from the cue position at longer latencies.

**Figure 4.**
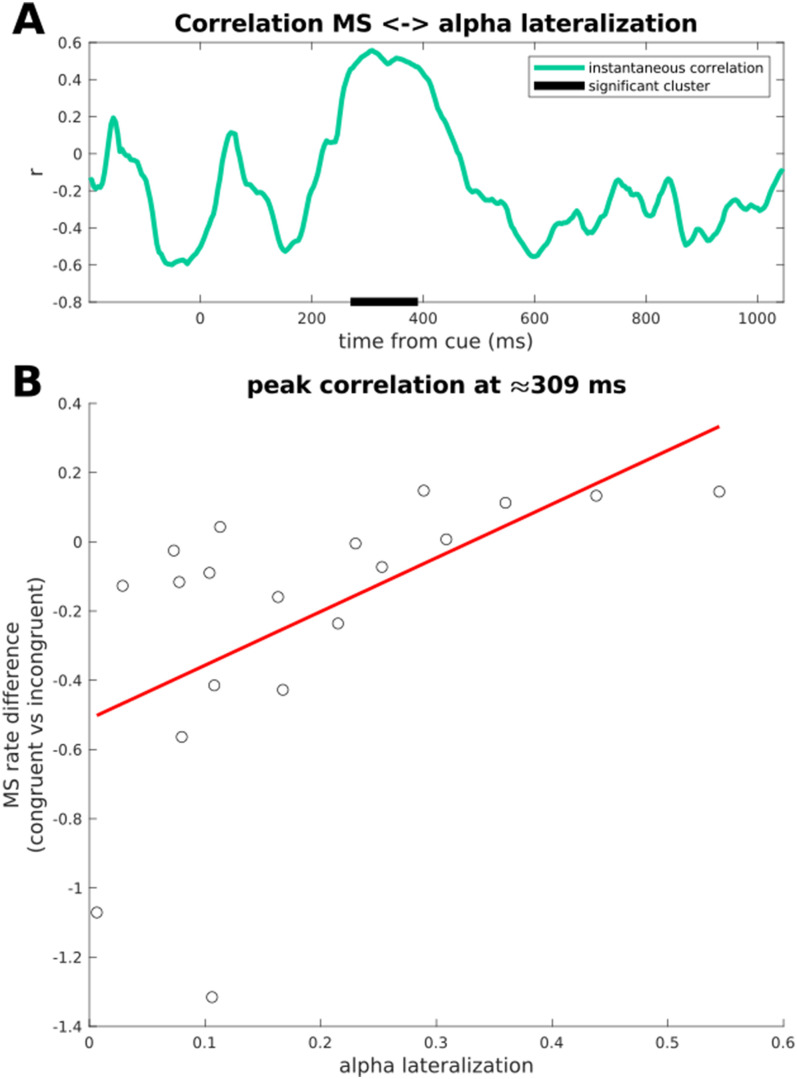
***A***, Correlation over time between alpha lateralization and MS-bias. The dark gray bar indicates the significant cluster after cluster permutation test. ***B***, Peak of the positive correlation at ∼309 ms.

To compare the pattern of alpha lateralization across time to the pattern of RTs across CTOAs, we averaged lateralization in no-target trials within seven time bins coinciding with the seven CTOAs in target trials. Thus, this sequence of lateralization averages in no-target trials reflects the momentary lateralization at the latencies when targets were presented in the different CTOA conditions, allowing for a direct comparison with the sequence of RTs at the corresponding CTOAs (Fig. 3*B*). While the RT time course showed behavioral facilitation at the cued location only at the earliest SOA (42 ms), alpha lateralization toward the cue continued to increase and peaked as late as 200 ms. *T* tests against zero performed on each CTOA for alpha lateralization confirm the pattern of results described beforehand, with the first CTOAs showing values significantly higher than 0 (CTOA = 42, *t*_(18) _= 8.576,  < 0.001; CTOA = 208, *t*_(18) _= 5.468, *p* < 0.001). Furthermore, while the RT time course showed a clear IOR effect at all but the earliest CTOA, alpha continued to be lateralized toward the cue until 375 ms (*t*_(18) _= 3.181; *p* = 0.005), and then returned to baseline (CTOA = 542; *t*_(18) _= 1.271; *p* = 0.219). Thus, while the IOR was in full swing, alpha lateralization did not show a corresponding reversal away from the cue, further confirmed by the last two CTOAs which did not diverge significantly from 0 (CTOA = 875, *t*_(18) _= −0.718, *p* = 0.482; CTOA = 1,042, *t*_(18) _= −0.677, *p* = 0.507).

We reasoned that the apparent mismatch between the time courses of RTs and alpha lateralization might have been influenced by interindividual variability in either variable. To address that, we conducted a correlation analysis to test if individuals with stronger behavioral IOR showed stronger lateralization in either direction. These correlations were computed between behavioral IOR effects at all CTOAs and lateralization at all latencies, resulting in a 7 × 7 correlation matrix. If stronger lateralization away from the cued location was associated with stronger behavioral IOR at the same time, this would show as a positive correlation in Figure 3*C*. If stronger lateralization was associated with IOR at a later time, this would show as a correlation above the diagonal. This analysis did not show any significant correlations along the diagonal (Fig. 3*C*). Moreover, although a few correlations were significant (e.g., positive correlations below the diagonal), none of them survived correction for multiple comparisons.

To sum up, alpha power was strongly lateralized toward the exogenous cue, with no evidence for a later re-lateralization away from the cue mirroring the strong behavioral IOR effect.

### Time course of microsaccades and its relation to RTs

We also examined the effect of exogenous cueing on MS-bias, computed as the difference between microsaccadic rate toward and away from the cued location. Again, this was based on the no-target condition. As for the alpha lateralization, we first tested MS-bias against 0 for the whole time window following the cue, revealing a directional bias away from the cued location (Fig. 3*D*), starting ∼250 ms, peaking at ∼370 ms, and fading away ∼600 ms (cluster *p* = 0.014). While the onset of this directional bias is compatible with the onset of IOR, MS-bias then fades away after 600 ms, whereas the IOR continues until after 1,000 ms.

To compare MS-bias to the pattern of RTs across CTOAs, we again averaged MS-bias in no-target trials within seven time bins coinciding with (i.e., just preceding) the seven CTOAs in target trials (Fig. 3*E*). This direct comparison shows an initial trend toward significance for MS-bias congruent to the cued location (CTOA = 208, *t*_(18) _= 1.812, *p* = 0.087), followed by MS-bias away from the cue at ∼400 ms, already shown by the cluster permutation analysis (CTOA = 375, *t*_(18) _= −2.39, *p* = 0.028; CTOA = 544, *t*_(18) _= −2.239, *p* = 0.038), to return to baseline in the last two CTOAs (CTOA = 875, *t*_(18) _= 0.377, *p* = 0.711; CTOA = 1,042, *t*_(18) _= −0.153, *p* = 0.883). The correlation between RTs and MS-bias (Fig. 3*F*) did not yield significant results.

### Correlation between MS-bias and alpha lateralization

At first glance, it seems that alpha lateralization and MS-bias showed diametrically opposite directions (Fig. 4): the pattern of microsaccades showed a bias away from the cued position, whereas alpha lateralization was directed toward the cued location, although these patterns show a remarkable temporal overlap. Accordingly, a point-by-point correlation across subjects between the two time courses yielded a cluster of positive correlation with onset at ∼270 ms and offset at ∼390 ms (cluster *p* = 0.008), peaking at ∼309 ms (*r* = 0.557; *p* = 0.013), indicating that subjects with stronger lateralization toward the cue had less MS-bias away from the cue.

### Bayesian analyses

**Figure 5.**
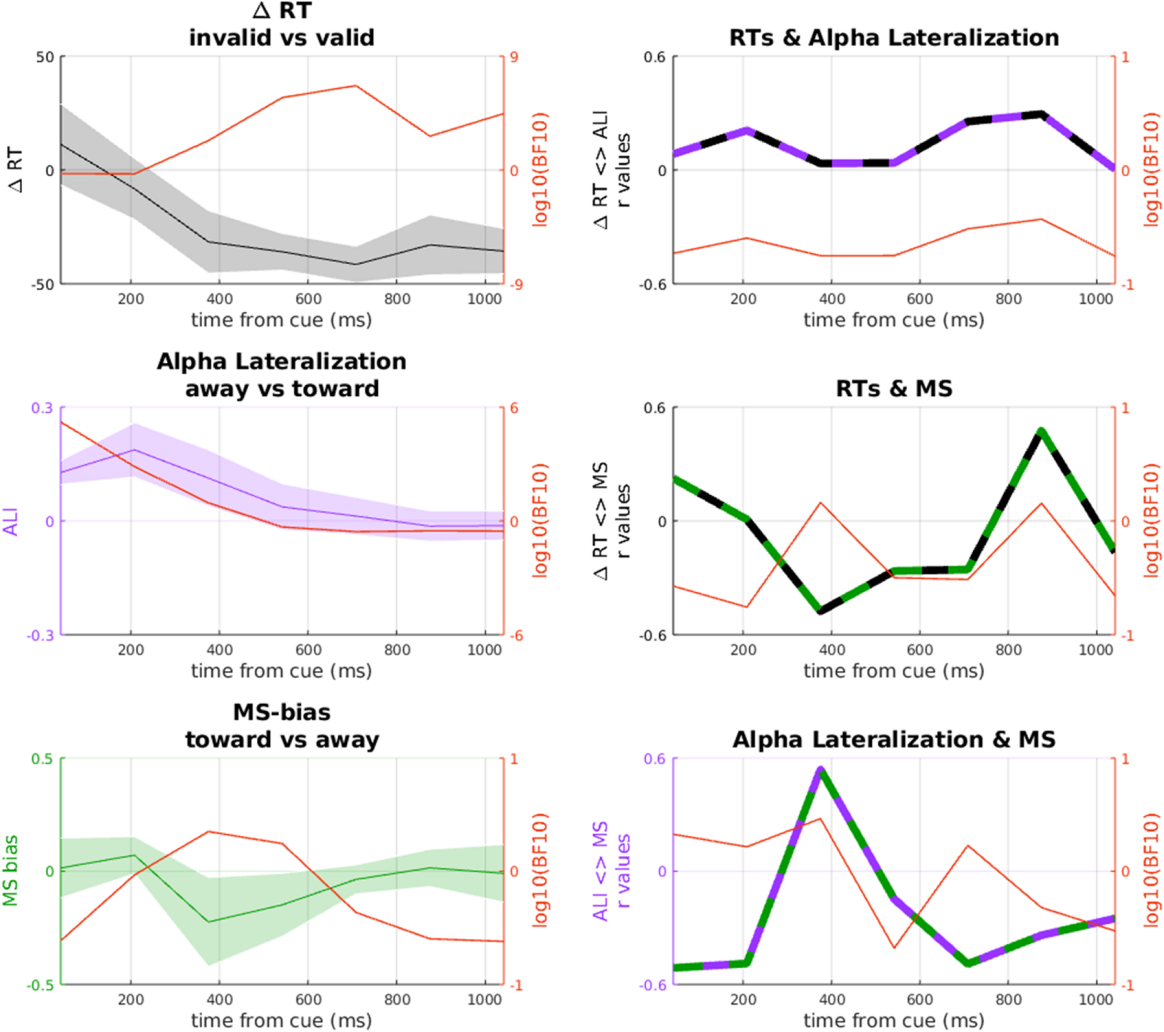
Bayesian analyses. The panels from ***A*** to ***C*** show the results from *t* tests against 0 for each of the measures of interests and each CTOA: RTs, alpha lateralization, and MS-bias. Shaded area represents 95% CI. The panels from ***D*** to ***F*** represent the results from the correlations, also for each CTOA. The paired variables are color coded in the dashed lines, with the same colors used in the left column. All the plots present a double *y*-axis: the measure of interest on the left axis and the corresponding log10 of Bayes factor on the right. For the latter, positive values show evidence in favor of H1, whereas negative values in favor of H0.

In order to provide a better characterization of RTs, alpha lateralization and microsaccades, especially in the light of the negative findings presented before, we complemented the previous set of analyses based on inferential statistics with a new set of analyses based on Bayesian statistics (see Materials and Methods). Firstly, we tested whether RTs, alpha lateralization, and MS-bias diverged from 0. For each CTOA, we computed Bayesian *t* tests against 0, to evaluate the amount of evidence in favor or against H0 (i.e., no difference from 0). Predictably, the RT time courses (Fig. 5) show extreme evidence of IOR for all the CTOA = 375 ms (CTOA = 375, $BF_{10}=209.24$; CTOA = 542, $BF_{10}=5.27\times 10^{5}$; CTOA = 708, $BF_{10}=4.7\times 10^{6}$; CTOA = 875, $BF_{10}=470.21$; CTOA = 1,042, $BF_{10}=2.88\times 10^{4}$), but anecdotal lack of evidence for an initial facilitation (CTOA = 42, $BF_{10}=0.51$). Alpha lateralization, coherently with previous inferential analyses, is strongly (CTOA = 42, $BF_{10}=1.63\times 10^{5}$; CTOA = 208, $BF_{10}=732$) or moderately (CTOA = 375, $BF_{10}=9.051$) directed toward the cue for the early CTOAs, but quickly converges to 0 in the late CTOAs: for all of these points; the Bayes factor suggest moderate evidence for the null hypothesis (CTOA = 708, $BF_{10}=0.27$; CTOA = 875, $BF_{10}=0.298$; CTOA = 1,042, $BF_{10}=0.291$). Also the MS-bias toward the cue shows a complex pattern but consistent with the one shown previously. It transitions from moderate-to-anecdotal evidence against an effect for the first and second CTOAs (CTOA = 42, $BF_{10}=0.243$; CTOA = 208, $BF_{10}=0.928$), to anecdotal evidence for a positive deflection for the second CTOA (CTOA = 208, $BF_{10}=0.928$), to anecdotal evidence for a negative deflection for the third and fourth CTOAs (CTOA = 375, $BF_{10}=2.241$; CTOA = 542, $BF_{10}=1.755$), to finally stabilize again around 0, with anecdotal (CTOA = 708, $BF_{10}=0.432$) and moderate evidence (CTOA = 875, $BF_{10}=0.253$; CTOA = 1,042, $BF_{10}=0.24$). As an interim conclusion, the Bayesian analyses confirm the strong effect of IOR, together with a marked alpha lateralization toward the cue, and a MS-bias away from the cue. Concurrently, they highlight the discrepancy between the IOR and alpha lateralization on one side and MS-bias on the other. In fact, both measures converge to 0 even at CTOAs at which the RT IOR effect is the most consistent. We further consolidate this result by examining the Bayes factors in the correlations between paired measures of interest. Starting from alpha lateralization and RTs, we find moderate evidence for a lack of effect spanning all the CTOAs (all $0.17<BF_{10}<0.31$) with the exception of anecdotal evidence in one CTOA (CTOA = 875, $BF_{10}=0.369$). The correlation between MS-bias and RTs also shows moderate lack of effect (all $0.17<BF_{10}<0.32$), with two exceptions (CTOA = 375, $BF_{10}=1.45$; CTOA = 875, $BF_{10}=1.43$), showing anecdotal evidence for a negative (CTOA = 375) and a positive (CTOA = 875) correlation. Finally, we assessed with Bayesian statistics also the correlation between MS-bias and alpha lateralization. Coherently with our previous analyses, we find anecdotal to moderate evidence of a positive correlation between the two phenomena at ∼375 ms from cue (CTOA = 375, $BF_{10}=2.915$). All the other CTOAs showed instead a negative correlation, with either anecdotal evidence for H1 $\left(1.69<BF_{10}<2.12\right)$ or anecdotal to moderate evidence for H0 (CTOA = 875, $BF_{10}=0.47$; CTOA = 542, $BF_{10}=0.21$; CTOA = 1,042, $BF_{10}=0.3$).

All in all, the analyses just presented corroborate the lack of relationship between alpha lateralization, MS, and IOR, whereas they provide anecdotal to moderate evidence of a correlation between MS and alpha lateralization.

### Single-trial regression between alpha lateralization and RTs

**Figure 6.**
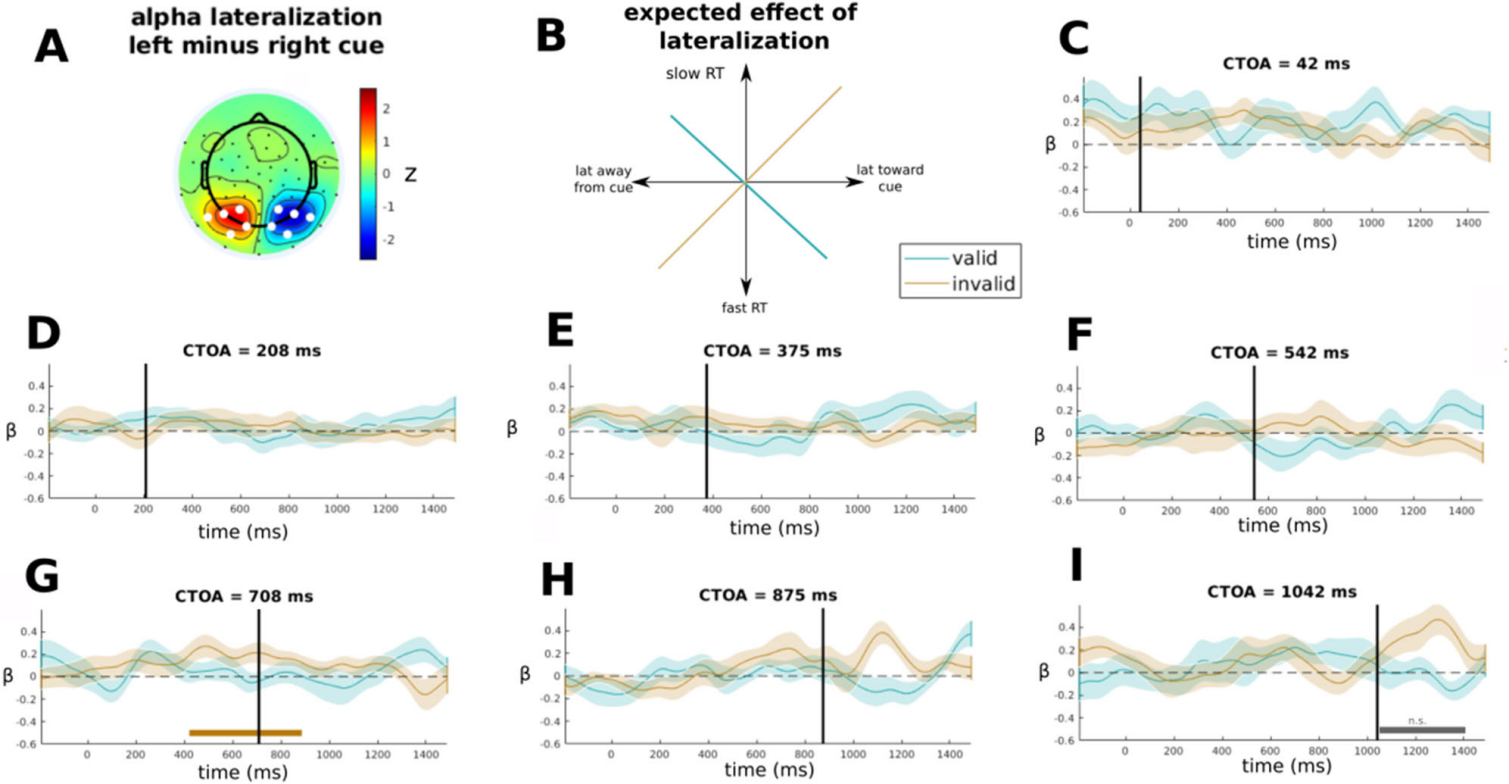
***A***, Average spatial filter used for EMS computation and based on the alpha lateralization pattern (left minus right cue) between 200 and 400 ms in the no-target condition. For the sake of comparison with the other analyses presented in the paper, white dots show the channels previously used for computing alpha lateralization. ***B***, Schematic representation of the expected effects for the single-trial regression between alpha lateralization and RT. The expected effects are presented separately for valid (negative slope) and invalid (positive slope). ***C–I***, Grand average of regression β scores computed in the single-trial regression analysis. Each panel shows a different CTOA condition, where the target onset is marked with a black vertical line. Shaded areas show SEM. Horizontal bars in the area below the graphs mark the presence either of a significant (colored bar) or marginally significant (gray bar) clusters.

In order to evaluate a plausible intraindividual relationship between the cue-induced lateralization and RTs, we computed a time-resolved regression analysis (see Materials and Methods). The results (Fig. 6) show that in the last three CTOAs for the invalid condition, the peri- or post-target lateralization index is positively associated with RTs. This effect is consistent across participants and leading to significant clusters at CTOA = 708 (cluster p=0.011) and to a marginally significant cluster at CTOA = 1,042 (cluster p=0.054). In other words, when a target is presented to the opposite side of the cue, a pattern of alpha lateralization that is more oriented toward the cue predicts higher (slower) RTs, for late CTOAs, although these effects do not survive multiple comparisons.

## Discussion

In the current study, we tested whether alpha lateralization tracks exogenous spatial attention, and specifically IOR in a typical exogenous Posner cueing task. For this purpose, we compared the time course of alpha lateralization after cue onset with the behavioral time course of IOR across seven CTOAs.

As expected, we found the typical IOR effect in behavior (Klein, 2000; Samuel and Kat, 2003) with facilitation at the cued position at short CTOA followed by strong and long-lasting inhibition of the cued position at long CTOAs (Fig. 1*B*). The IOR is probably not a unitary phenomenon: depending on the specifics of the task and paradigm, it may result from input-based (i.e., sensory, attentional) or output-based (oculomotor) mechanisms (Berlucchi, 2006; Dukewich and Klein, 2015). The experimental paradigm used in the present study, specifically the use of manual responses and tonic suppression of saccades, has been demonstrated to elicit predominantly input-based IOR effects (Klein, 2000; Taylor and Klein, 2000; Klein and Hilchey, 2011; Redden et al., 2021). For instance, several studies have demonstrated that, under these conditions, IOR affects not only RTs but also visual sensitivity (Handy et al., 1999; Ivanoff and Klein, 2006). Furthermore, McDonald et al. (2009) investigated the N2pc (an event-related potential indicating a shift of attention) in an adaptation of the classical IOR paradigm and found that the N2pc was reduced for targets presented at recently attended locations. When no target appeared, the N2pc was even directed away from the previously attended location, thus confirming an attentional mechanism. Thus, we conclude that the behavioral IOR effect found in the present study reflects initial attentional orienting toward the cued position, followed by reorienting of attention away from and inhibition of the cued location.

Given the hypothesized role of alpha lateralization in modulation of input gating under endogenous (Foxe and Snyder, 2011) and exogenous attention (Feng et al., 2017; Keefe and Störmer, 2021), we expected that alpha lateralization would show a time course and direction consistent with the time course of behavioral effects: an increase in alpha ipsilaterally and a decrease contralaterally to the cue at early latencies (<225 ms after cue onset), followed by a reversed lateralization pattern at longer latencies. Indeed, we observed an initial, strong alpha lateralization toward the cue, in agreement with previous studies (Feng et al., 2017; Keefe and Störmer, 2021; Fig. 2). Nonetheless, this lateralization emerged only after behavioral facilitation had already disappeared (Fig. 3*B*), implying that alpha lateralization had no direct causal role for producing the behavioral cueing effect. This result resembles that of Antonov et al. (2020) who found that the effect of endogenous spatial cues on alpha lateralization lagged behind the cueing effect on behavioral performance. Similarly, Bacigalupo and Luck (2019) found that alpha lateralization in a visual search task outlasted average RTs and other electrophysiological indices of attention.

Moreover, there was no subsequent re-lateralization of alpha power (i.e., decrease ipsilateral/increase contralateral to cue) at latencies longer than 225 ms mirroring the behavioral IOR effect. This missing re-lateralization in the face of a strong behavioral inhibition effect is striking given the alleged role of alpha oscillations in attentional inhibition (Foxe and Snyder, 2011). Nonetheless, several other studies have also found no evidence for a direct role of alpha lateralization for inhibition of noncued or irrelevant locations or stimuli (Noonan et al., 2016; Slagter et al., 2016; Antonov et al., 2020; Gundlach et al., 2020). Our single-trial analysis (Fig. 6) shows some weak relationship between moment-to-moment variations of cue-induced alpha lateralization and RTs, suggesting that when alpha lateralization is anchored to the cued position, the participants respond slowly to stimuli in the uncued position. Intriguingly, this effect is concentrated on the last CTOAs, when the IOR effect is the most prominent. Nonetheless, this effect was not strong enough to survive multiple comparisons and present only in one validity condition, casting doubts on its reliability and reproducibility. Altogether, this evidence contributes to the growing body of literature suggesting that the suppressive account of alpha oscillations might have to be revisited (Slagter et al., 2016; Foster and Awh, 2019; van Moorselaar and Slagter, 2020). To conclude, the delayed emergence of alpha lateralization toward the cue and the missing subsequent re-lateralization mirroring the behavioral IOR suggest that cue-induced alpha-band lateralization may not be directly involved in producing behavioral effects of exogenous spatial orienting or in modulating the neural response to target stimuli. Rather, alpha lateralization might be associated with other processes coinciding with cueing, or with salient events in general, such as saccadic eye movements.

While subjects generally followed the instruction to maintain steady central fixation, they frequently made miniature eye movements (<1 dva), and the direction of these microsaccades was consistently biased away from the cued location. This bias is probably a consequence of the inhibition of larger, reflexive saccades toward the exogenous cue (Rolfs et al., 2004). Notably, this compensatory microsaccade bias peaked at about the same time when alpha was maximally lateralized *toward* the cued location (Fig. 3*A*,*D*). Even though these effects were tuned in opposite directions, they were positively correlated: stronger alpha lateralization toward the cue was strongly associated with *reduced* microsaccade bias away from the cue. Although this effect was confirmed by an additional Bayesian analysis, it has to be acknowledged that the magnitude of this effect was anecdotal to moderate. Therefore a further replication, with a larger sample size, would be necessary to substantiate this effect.

Nonetheless, the relationship between saccadic and microsaccadic eye movements and the alpha rhythm has long been established (Gaarder et al., 1966; Ulrich, 1990; Dimigen et al., 2009; Staudigl et al., 2017). Recently, interest in this relationship has been renewed based on studies showing that cue-induced alpha lateralization is stronger in trials with cue-driven microsaccades (Liu et al., 2022a) and that microsaccades are followed by alpha lateralization transients (Liu et al., 2022b). Importantly, Liu et al. (2022a) observed cue-induced alpha lateralization even in the absence of a microsaccade, suggesting that alpha lateralization is associated with both executed as well as subthreshold, nonexecuted microsaccades, thus representing two aspects of oculomotor behavior (Liu et al., 2022a). In a similar vein, Popov et al. (2021) have demonstrated that alpha power modulations are strongly coupled with eye movements and that the scalp topographies of saccade-related alpha power modulations are consistent with saccade direction. Given that eye movements are often biased toward cued or task-relevant locations, and given that miniature eye movements can easily escape fixation monitoring and EEG artifact removal, this may explain the high topographical specificity of alpha topographical distributions in tasks using spatial cueing (Rihs et al., 2007), visual search (Samaha et al., 2016; Foster et al., 2017), and visual working memory (Foster et al., 2016). Hence, we propose that cue-induced alpha lateralization in this study did not reflect a mechanism subserving the modulation of neuronal responses to target stimuli, but a function of the oculomotor system. Given that task instructions demanded steady fixations, pro-saccades had to be suppressed, as indicated by microsaccade bias in the opposite direction. The positive correlation between alpha lateralization and microsaccade bias (i.e., reduced bias away from the cue when alpha lateralization toward the cue was strong) suggests that both phenomena reflect the preparation for nonexecuted, reflexive pro-saccades toward the exogenously cued location.

This oculomotor interpretation is partially consistent with previous accounts of the role of alpha oscillations for cortical excitability and inhibition (Klimesch et al., 2007; Jensen and Mazaheri, 2010; Foxe and Snyder, 2011) by assuming that alpha-band lateralization is involved in controlling excitability in anticipation of saccade execution and landing. Given the tight coupling between eye movements and shifts of attention, this involvement would be consistent with the effect of spontaneous fluctuations of alpha-band lateralization and spatial attention (Bengson et al., 2014) or subjective contrast appearance (Balestrieri and Busch, 2022). Moreover, given that most cueing paradigms are designed such that attention is oriented toward the cued location, cue-induced alpha lateralization will typically show lateralization toward the attended location, even though it may not directly serve the neural amplification of attended targets or inhibition of unattended input. The IOR paradigm, in which attention is oriented away from the cued location at longer latencies, makes it possible to reveal that alpha lateralizes toward the salient cue stimulus, not toward the direction of attentional (re)orienting.

## Conclusion

The current study shows that alpha lateralization does not track the time course and direction of attentional orienting in the IOR paradigm with exogenous cues. However, the time course of alpha lateralization toward the cue was very similar to that of microsaccades away from the cue.

This set of results, together with recent evidence from the literature, suggests that alpha lateralization and microsaccades are both consequences of planned, but ultimately suppressed, saccades toward the cued position. In this framework, alpha lateralization would modulate cortical excitability in anticipation of the saccade itself, whereas the microsaccades in the direction opposite to the cue would be reactively generated by suppressing the saccade toward the cue. This claim is further strengthened by a covariation observed between the two variables: the stronger the microsaccadic rate away from the cue, the weaker the alpha lateralization toward the cue.

## Data Availability

It is possible to access the pre-registration of the present study at the following link: https://aspredicted.org/blind.php?x = MWH_Y88. Data and all the code for the present study are provided through following OSF link: https://osf.io/8t963/.

## Synthesis

Reviewing Editor: Anne Keitel, University of Dundee Decisions are customarily a result of the Reviewing Editor and the peer reviewers coming together and discussing their recommendations until a consensus is reached. When revisions are invited, a fact-based synthesis statement explaining their decision and outlining what is needed to prepare a revision will be listed below. The following reviewer(s) agreed to reveal their identity: Christopher Benwell.

Both reviewers and the editor agreed that this was an important and well done study and the assessment was overall very positive. However, a few issues were brought up by the reviewers, which are added below. The comments can be summarised to the following main points:

1) The sample size of N=19 is borderline acceptable from the viewpoint of current best practices. The low sample size and potential consequences for statistical power and interpretation should be discussed transparently.

2) Reviewer #1 suggested performing an additional trial-by-trial analysis to gauge whether measures are associated with within-participant variations, which Reviewer #2 and the editor agreed would be a very useful addition to the manuscript.

3) Some more information to assess the data would be helpful (additional topographies, CI values) to better interpret the results. During the discussion, the idea to use Bayesian analysis for the correlations also came up, which could aid interpretation of the null effects.

You will find these main issues as well as other comments in the unabridged reviewer comments below. Please address all comments in a point-by-point manner.

*****

Reviewer #1 - Advances the Field (Required)

The authors leveraged a classical spatial attention paradigm and were able to show a common signature of spatial attention selection, i.e. alpha-band activity, and found that alpha-band activity did not follow the attentional dynamics derived from behavioral measures.

Reviewer #1 - Comments to the Authors (Required)

Alpha-band lateralization and microsaccades elicited by exogenous cues do not track attentional orienting

The authors aimed at examining the role of alpha-band activity in spatial attention. They specifically leveraged the Inhibition of Return phenomenon associated with a dynamic shift of spatial attention first towards and then away from an exogenously cued spatial position to study whether the dynamics of EEG recorded alpha band-activity followed these dynamics. While recorded reaction times to target stimuli presented at varying intervals after the cue showed the expected IOR pattern, alpha band activity was only lateralized towards the cued side and never in an opposite direction. Recorded micro-saccade patterns were associated with these alpha-band dynamics letting the authors conclude that for this experimental paradigm, alpha-band activity rather seems to represent a mechanism associated with oculomotor control. This is discussed in light of similar findings in recent as well as older literature. I overall found the experiment, analysis, and manuscript quite interesting and convincing, and just have a few points that may need to be clarified.

First, one major part of the analysis and argumentation relies on the interpretation of null effects. The absence of significance does, however, not necessarily imply the absence of an effect. Potential effects may just stay unnoticed due to low power for instance. This may be especially the case for the correlation analyses (see Button et al., 2013, Nature Reviews Neuroscience; see also Yarkoni, 2009, Perspectives in Psychological Science). These analyses are based on 19 data points (i.e. the number of subjects). As calculated with GPower, correlation coefficients of 0.56 or larger (or -0.56 or smaller) would be required to be measurable with a power of 0.8 and an alpha error of .05 for a sample of 19 participants. So for relatively low numbers of participants, it is often recommended to interpret the point estimation of the correlation coefficients with caution and to include confidence intervals for these.

Related to the correlational analysis, the correspondence measures between alpha-band activity, microsaccades, and reaction times are based on potentially corresponding between-subject variance (or a lack thereof), i.e. subjects with higher alpha lateralization should also show higher RT differences. While the authors find no between-subject effects, alpha-band reaction time correspondences may be found intra-individually: alpha-band lateralization magnitudes interindividually varying on a trial-by-trial level may be systematically related to reaction time variations, as frequently found when relating pre-target alpha-band amplitudes and behavioral measures. Do the authors see any possibility of analyzing alpha-band, reaction, and microsaccade correspondences on a trial-by-trial level?

############

Minors:

• Line 29: "substrate for attentional" - "substrate of attentional"

• Line 63: "in response of spatial cue" - "in response to a spatial cue"

• Line 208: How was the smoothing implemented? Kalman filter, median filter, ...

• Line 229/230: I don't understand which analysis does this point to? And what does it mean to use "original data" in contrast to the analysis described in the paragraph prior to line 229.

• Line 242: What is meant by contiguous significant points: in a temporal sense or also spatial sense, i.e. stemming from related electrodes?

• Line 292: To me the description rather points to Figure 3A?

############

References

Button, K. S., Ioannidis, J. P. A., Mokrysz, C., Nosek, B. A., Flint, J., Robinson, E. S. J., et al. (2013). Power failure: why small sample size undermines the reliability of neuroscience. Nat. Rev. Neurosci. 14, 365-376. doi:10.1038/nrn3475.

Yarkoni, T. (2009). Big Correlations in Little Studies: Inflated fMRI Correlations Reflect Low Statistical Power-Commentary on Vul et al. (2009). Perspect. Psychol. Sci. 4, 294-298. doi:10.1111/j.1745-6924.2009.01127.x.

Reviewer #2 - Advances the Field (Required)

The study convincingly shows that neither post-cue EEG alpha lateralization nor biases in lateralized micro-saccades track shifts in exogenous attentional capture as indexed by the inhibition-of-return (IOR) phenomenon.

Reviewer #2 - Comments to the Authors (Required)

The authors tested whether post-cue lateralized alpha EEG activity and/or lateralized micro-saccades represent physiological substrates of the inhibition of return (IOR) phenomenon. Behaviourally, the experimental task induced the typical IOR effect as expected. However, though the cue induced reliable lateralization alpha power, this lateralization did not track the IOR phenomenon. Lateralized micro-saccades also didn't convincingly track the IOR effect. Interestingly, the alpha and micro-saccade lateralization effects were positively correlated. The authors suggest that the results do not support lateralized alpha power as playing a direct role in exogenous attentional capture, but rather interpret the effects to support an oculomotor account of the function of cue-induced alpha lateralization.

The study is technically sound, rigorous, and interesting. The manuscript is well written, and the results support the authors interpretations. It is commendable that the authors pre-registered the study and will make the data publicly available. I only identified minor issues that the authors may wish to address:

1. Regarding the between-subject correlation between alpha lateralization and micro-saccade bias direction, this result is interesting (and worth reporting) but I would urge the authors to adopt caution in the interpretation here. Given the sample size of 19 is very small for conducting a between-subjects correlation, it might be worth acknowledging this limitation in the discussion. The authors already acknowledge this analysis was not pre-registered and I think replication with a larger sample would be necessary to make any strong interpretations here.

2. It would be helpful to show a topographic representation of the cue-induced alpha lateralization (maybe just for the no target condition) just as a sanity check that the chosen regions of interest were appropriate here, given that they were chosen based on a previous study.
